# Thermostability Improvement of the Chitinase from *Bacillus circulans* for Efficient Chitin Oligosaccharide Production via Computational Design

**DOI:** 10.3390/biom15030330

**Published:** 2025-02-24

**Authors:** Jingwei Liu, Jie Xie, Si Wang, Hong Feng, Ganggang Wang

**Affiliations:** 1 Key Laboratory of Environmental Microbiology of Sichuan Province, Chengdu Institute of Biology, Chinese Academy of Sciences, Chengdu 610041, China; liujw@cib.ac.cn (J.L.); xiejie@cib.ac.cn (J.X.); wangsi@cib.ac.cn (S.W.); 2 Key Laboratory of Environmental and Applied Microbiology, Chengdu Institute of Biology, Chinese Academy of Sciences, Chengdu 610041, China; 3 College of Life Sciences, Sichuan University, Chengdu 610064, China; hfeng@scu.edu.cn; 4 University of Chinese Academy of Sciences, Beijing 100049, China

**Keywords:** chitinase, thermostability, computational design, chitin oligosaccharides, protein engineering

## Abstract

The chitinase A1 from *Bacillus circulans* WL-12 (BcChiA1) exhibits promising potential for producing chitin oligosaccharides (CHOs), while its application is limited by its poor thermal stability. In this study, a set of thermostable variants were obtained by modifying BcChiA1 using a comprehensive strategy based on a computer-aided design. A combination of five beneficial single-point mutations (S67G/K177R/A220V/N257Y/N271E) to BcChiA1 generated a markedly improved variant, Mu5. Mu5 exhibited a half-life of 295 min at 60 °C, which was 59 times higher than that of BcChiA1. Furthermore, Mu5 was reused for chitin conversion, releasing 86.14 ± 3.73 mM of CHOs after five reaction cycles. Molecular dynamics simulation and structural analysis revealed that these enhancements were driven by increased structural rigidity and compactness, resulting in a protein conformation that was less prone to thermal denaturation. This combined approach through computational design yielded a thermostable BcChiA1 variant, potentially facilitating CHOs production in economical way.

## 1. Introduction

Chitin consists of β-(1,4)-linked N-acetyl-D-glucosamine (GlcNAc) units and is the second most abundant biomass after cellulose. The global annual production of chitin is about 100 billion tons, which is a renewable biomass resource [1,2,3]. Although chitin is abundant in resources, its high crystallinity and low solubility limit its practical application [4,5]. Chitin oligosaccharides (CHOs), produced by the hydrolysis of chitin, exhibit a range of biological activities, including antioxidant, antibacterial, immunomodulatory, and anti-tumor properties. CHOs could be widely used in food, medicine, agriculture, and other industries [6,7,8]. Therefore, the efficient production of high-quality CHOs from chitin is a crucial goal in the industrial field.

Currently, the CHOs are primarily produced by chemical or enzymatic methods. The chemical hydrolysis of chitin involves large amounts of strong acids and bases, which are harmful to the environment. Additionally, these chemical approaches are a form of non-selective degradation, generating CHOs with varying degrees of polymerization. In contrast, the enzyme-catalyzed depolymerization of chitin could produce specific CHOs in a controlled manner [9,10]. This selective process not only reduces the environmental risks but also enhances the precision of the product.

Chitinase (EC 3.2.1.14) is a key hydrolase that can specifically convert chitin into CHOs [11]. However, owing to the poor stability of the enzymes, most native chitinases cannot retain catalytic activity during a prolonged reaction time, not to mention the reusability [12,13,14]. Therefore, enhancing the thermal stability of chitinase is crucial to improve its potential for industrial applications. A common approach is to increase the rigidity of flexible regions within the enzyme to enhance its thermal stability [15,16]. The thermal stability of chitinase from *Serratia marcescens* B4A was improved by replacing glycine 191 with valine in a surface flexible loop; the half-life (*t*_1/2_) of the G191V variant increased 15-fold at 60 °C [17]. In *Paenibacillus pasadenensis* CS0611, the stability of the chitinase was enhanced through a semi-rational design and the introduction of a disulfide bond, resulting in a variant with a 26-fold increase in half-life at 50 °C [18]. Although the thermal stability of chitinase has been effectively improved through mutations in surface flexible loops and the introduction of disulfide bonds, a more comprehensive strategy is still needed to further optimize the enzyme’s performance in industrial applications. Recent advances in protein structure prediction and rational design have greatly facilitated the identification of stable variants. Methods such as co-evolutionary analysis, energy function calculations, and phylogenetic analysis have been applied in predicting mutations with enhanced stability [19,20,21]. To date, the computational design of protein has evolved into a systematic approach for engineering proteins with specific functions [22,23,24].

The chitinase A from *Bacillus circulans* WL-12 (BcChiA1) exhibits excellent hydrolytic activity on the chitin, while its application is limited by its poor thermal stability and nonreusability [25,26]. In this study, three computational methods (ABAUCS, PROSS, and FireProt 2.0) were applied to identify potential variants in BcChiA1. Through this comprehensive approach, a set of single variants were generated, and most of them showed improved thermostability. Finally, a combinational variant, Mu5 (S67G/K177R/A220V/N257Y/N271E), was successfully constructed, which showed enhanced thermal stability and excellent performance in the continuous hydrolysis of colloidal chitin. Molecular dynamics (MD) simulations and structural analysis revealed that the enhanced thermal stability of the variant Mu5 primarily resulted from the increased structural rigidity and compactness. Through this comprehensive design strategy, a robust chitinase variant was generated, which will be beneficial for CHO production in an economical way.

## 2. Materials and Methods

### 2.1. Chemicals and Materials

Chitin was purchased from Sigma-Aldrich (Shanghai, China) and the chitooligosaccharide standards were supplied by Qingdao BZ Oligo Biotech Co., Ltd. (Qingdao, China). *E. coli* DH5α and *E. coli* BL21(DE3) cells were acquired from Novagen  (Beijing, China), whereas the pET-28a vector was purchased from Thermo Fisher Scientific (Shanghai, China). All of the PCR reagents and enzymes were supplied by TaKaRa (Dalian, China). Primers were synthesized at Sangon Biotech (Shanghai, China). The remaining unlisted chemical reagents were analytically pure.

### 2.2. Construction of BcChiA1 and Variants

The gene (Genbank accession number: AAA81528.1) encoding BcChiA1 (excluding the N-terminal signal peptide) was synthesized by Tsingke Biotech Co., Ltd. (Hangzhou, China). The gene was cloned into a pET-28a vector and the constructs (with an N-terminal His-tag) were verified by DNA sequencing. Mutations were performed using the QuikChange method [27] with the plasmid pET28a-BcChiA1 as the template, employing the specific primers listed in Table S1.

### 2.3. Computational Prediction for Chitinase Thermostability

Based on the crystal structure and sequence of the catalytic domain of BcChiA1 (PDB ID: 1ITX [28]), the selection of PROSS, FireProt 2.0, and ABAUCS for thermal stability optimization leverages their complementary strengths. PROSS combines conformational free energy calculations and sequence conservation analysis, enabling large-scale optimization by identifying stability-enhancing mutations [29]. FireProt 2.0 integrates evolutionary, structural, and energetic data, providing a comprehensive evaluation of the mutation effects on the stability [30]. ABAUCS focuses on adaptive backbone remodeling and thermodynamic calculations, excelling in optimizing the stability for complex proteins [31]. Based on the outcome of these three software programs, the AlphaFold2 database (https://www.alphafold.ebi.ac.uk/, accessed on 21 March 2024) was employed to predict the structure of each variant. Unless otherwise specified, all of the predictions were performed using the default settings. To enhance the accuracy of the structural models, a template-based prediction approach was employed, utilizing the structure of the catalytic domain of BcChiA1 as the template. The final predicted models were subjected to the relaxation process by using the default relaxation protocol provided in AlphaFold2 so to optimize the geometry and correct potential steric clashes. Subsequently, the interactions between amino acid residues before and after mutation were compared using PyMOL 2.5 (https://pymol.org/ accessed on 26 March 2024), further analyzing the potential effects of the mutations on the protein stability.

### 2.4. Expression and Purification of BcChiA1 and Its Variants

The pET28a-BcChiA1 plasmid was transformed into *E. coli* BL21 (DE3). The *E. coli* BL21 (DE3) cells were cultured in LB medium containing 60 µg/mL of kanamycin (kana) at 37 °C until OD600 reached 0.6–0.8. Then, 1 mM isopropyl-β-dithiogalactopyranoside (IPTG) was added to the culture medium for induction and incubated at 16 °C for 12 h. After cultivation, the cells were collected via centrifugation (10,000× *g*, 10 min, and 4 °C). The harvested cells were resuspended in the lysis buffer (50 mM Tris-HCl, 500 mM NaCl, and a pH of 8.0) and subjected to sonication in ice water to facilitate cell lysis. After centrifugation (10,000× *g*, 30 min, and 4 °C), the supernatant was applied to a Ni-NTA affinity chromatography column equilibrated with buffer, and the target protein was collected using an elution solution containing 150 mM imidazole. After purification, the protein was immediately dialyzed to effectively reduce the concentration of imidazole. The purified protein sample was analyzed by SDS-PAGE and the protein concentration was determined using the Bradford method [32]. Production of variant proteins was carried out using the same protocol used to purify the wild-type BcChiA1.

### 2.5. Enzyme Activity Measurement

An evaluation of the enzyme activity was carried out using colloidal chitin as a substrate. Colloidal chitin was prepared as previously described with some modifications [33]. The chitinase activity was evaluated using the modified 3,5-dinitrosalicylic acid (DNS) method [34] with D-glucosamine as a standard. An amount of 10 µL of diluted enzyme solution was added into to 290 µL of 1% (*w*/*v*) colloidal chitin solution. The reaction was incubated at 50 °C for 10 min and terminated by boiling in water for 5 min. Subsequently, the reducing sugar from the reaction was measured using the DNS method. Under the above conditions, the amount of enzyme required to produce 1 µmol of reducing sugar per minute was defined as one unit of chitinase activity. All of the assay reactions were carried out in triplicate.

### 2.6. Determination of Thermal Stability of BcChiA1 and Its Variants

To investigate thermal stability of BcChiA1 and its variants, their residual enzyme activities were measured. The BcChiA1 and variants were incubated at 55 °C for 15 min, immediately cooled, and then centrifuged to remove the precipitant. Subsequently, the enzyme activity of the supernatant was measured under the standard assay conditions.

The half-life (*t*_1/2_) of an enzyme is defined as the time required for it to lose 50% of its activity under a specified condition. In this study, BcChiA1 and its variants were incubated at 60 °C for different durations. The residual activity of the enzymes was measured to calculate the half-life.

The melting temperature (*T*_m_) of proteins is an important indicator for measuring the thermodynamic stability of enzymes, reflecting the conformational changes that occur under high-temperature conditions [35]. In this study, the *T*_m_ value of the enzyme was determined by the differential scanning fluorescence (DSF) method. The specific operation was performed on a CFX 96 real-time PCR instrument (Bio Rad, Hercules, CA, USA) at an excitation wavelength of 490 nm and an emission wavelength of 575 nm. Firstly, the purified enzyme was diluted to the appropriate working concentration and mixed with SYPRO orange dye. Subsequently, the mixture was divided into 96-well PCR plates and subjected to temperature scanning, with a temperature range of 20 °C to 95 °C and a heating rate of 1 °C per minute. During the entire temperature scanning process, real-time changes in fluorescence signals were monitored to determine the melting temperature of the proteins. All of the measurements were performed in triplicate.

### 2.7. Enzymatic Property Analysis and Kinetic Parameter Determination

To evaluate the optimal reaction temperature of the enzyme, the specific activity of the wild type and variant Mu5 was measured at temperatures of 20, 30, 40, 45, 50, 55, 60, and 70 °C. By comparing the specific activity data at different temperatures, the optimal reaction temperature for the two enzymes was determined.

To determine the kinetic parameters, the purified enzyme was mixed with colloidal chitin solutions of different concentrations (2, 4, 6, 8, and 10 mg/mL, respectively) in the experiment. Under the optimal reaction conditions, the enzyme reaction lasted for 10 min. Subsequently, the Michaelis constant (*K*_m_) and catalytic constant (*k*_cat_) were calculated using the GraphPad Prism 8 software.

### 2.8. Hydrolytic Properties of the BcChiA1 and Its Variants

To measure the conversion of the substrate, the purified enzyme (5 mg/mL) was mixed with 1% (*w*/*v*) colloidal chitin solution (300 µL) and incubated at 50 °C. At specified time intervals, samples were collected and the reducing sugars in the hydrolysate were quantified using the DNS method. The substrate conversion rate was calculated based on the molecular weight of chitobiose.

To assess the reusability of variant Mu5, 5 cycles of substrate conversion and product accumulation were carried out in the same reaction system. In each cycle, the colloidal chitin was hydrolyzed for 90 min at 50 °C and the amount of CHO products was measured; then, an equivalent amount of fresh colloidal chitin was added for the next cycle.

BcChiA1 and the variant Mu5 were incubated with colloidal chitin at 50 °C for 90 min; then, the reaction was stopped by boiling for 5 min. After centrifugation, the supernatants of the mixtures were concentrated by about 10 times. Subsequently, the degradation products were analyzed by using thin-layer chromatography (TLC). The solvent system comprising n-butyl alcohol, methanol, 28% ammonia solution, and water (10:8:4:2, *v*/*v*) was utilized. After development, the plates were treated with an aniline–diphenylamine reagent (4 mL of aniline, 4 g of diphenylamine, 200 mL of acetone, and 30 mL of 85% phosphoric acid), and then baked at 120 °C until spots appeared.

### 2.9. Molecular Dynamics (MD) Simulations

The ligand structure was sourced from the complex structure of PDB databases (PDB ID: 3B9A [36]). The variant Mu5 was modeled using the AlphaFold2 database with the structure of the catalytic domain ofBcChiA1 (PDB ID: 1ITX) as a template. Molecular docking was performed using AutoDock 4.2 to investigate the binding interactions between the enzyme and its substrate [37]. Prior to docking, the protein and ligand structures were optimized by assigning Gasteiger charges and defining the rotatable bonds to allow for conformational flexibility. The docking simulations were conducted using the Lamarckian Genetic Algorithm. A grid box was centered on the predicted active site of the enzyme, encompassing all residues within a 10 Å radius of the catalytic center to ensure the comprehensive coverage of potential binding regions. The evaluation of the docking results was based on binding energy scores. To validate the reliability of the docking results, a structural comparison was performed using the crystal structures of *Vibrio harveyi* chitinase A (PDB ID: 3B9A) [36] as a reference model. The analysis focused on the alignment of key active site residues and the consistency of ligand-binding modes to assess the structural agreement between the docked and experimentally resolved complexes. The binding conformations with the lowest binding energies and favorable interactions were selected for further analysis, such as hydrogen bonds and hydrophobic contacts with catalytic residues.

Prior to conducting the MD simulations, a series of preparation steps were carried out to ensure the structural stability and reliability. Initially, the ligand was subjected to energy minimization using the MMFF94 force field in ChemDraw (version 2022.1) to optimize the molecular conformations and eliminate unfavorable geometric configurations. Subsequently, both ligand and protein models were imported into GROMACS (version 2019.3)  for further refinement [38]. The AMBER99SB-ILDN force field and GAFF force field were utilized for the parametrization of the protein and ligand, respectively [39,40]. The protein–ligand complex was embedded in a dodecahedral box with periodic boundary conditions and solvated using the TIP3P water model. To maintain charge neutrality, Na⁺ ions were added to the system. Energy minimization was then performed using the steepest descent algorithm with a maximum step size of 0.01 nm and an upper limit of 50,000 steps. This iterative process aimed to reduce steric clashes and relieve local structural strain until the system reached energy convergence, thereby ensuring structural stability. Following energy minimization, the system underwent equilibration through a two-step process. First, a 1000 ps NVT simulation was conducted to stabilize the system under constant volume and temperature conditions at 338 K. This was followed by a 1000 ps NPT simulation to equilibrate the system under constant pressure and temperature conditions. These preparatory steps ensured that the initial structures were well-equilibrated, providing a stable foundation for the subsequent MD simulations and enhancing the reliability of the computational analyses. Finally, the work was submitted to the Beijing Super Cloud Computing Center (BSCC) for 100 ns production MD simulations, with the simulation time step set to 2 fs and traces saved every 100 ps. By analyzing the atomic trajectories, data on the root mean square deviation (RMSD), solvent-accessible surface area (SASA), root mean square fluctuation (RMSF), and radius of gyration (*R*_g_) were generated. The initial energy-minimized structure of each system was used as the reference structure for the RMSD calculations to accurately assess conformational deviations during the production MD simulations. The structural analysis and visualization were carried out by PyMOL.

## 3. Results and Discussion

### 3.1. Design and Screening of Single-Point Variants

**Figure 1:**
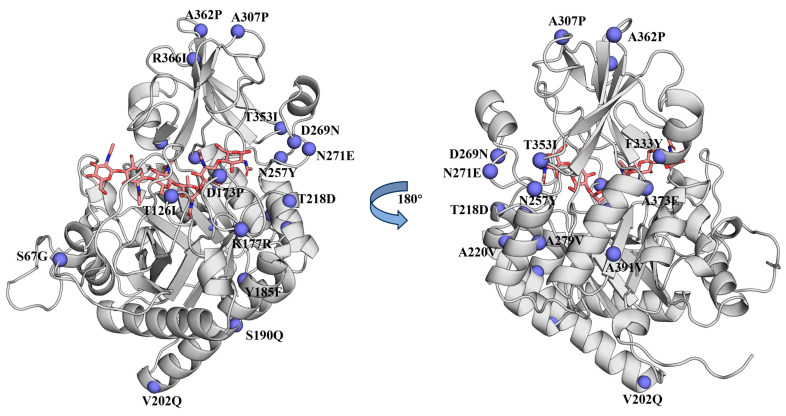
Three-dimensional (3D) model of the catalytic domain of BcChiA1 and the candidate sites for variation. In the BcChiA1 model, violet spheres represent the candidate sites, and the substrate is shown with pink sticks.

To explore strategies for improving the thermal stability of BcChiA1, computational design was conducted using three advanced algorithms. A total of 31, 13, and 32 potential variants were identified by PROSS, FireProt 2.0, and ABAUCS, respectively (Table S2). By leveraging the complementary strengths of these algorithms, a multi-dimensional analysis was performed, including sequence diversity, structural robustness, and evolutionary adaptability. Recognizing the need to balance variant diversity and experimental feasibility, a stringent selection framework was employed based on the following three pivotal exclusion criteria [19,22]: (i) the sites proposed by only one algorithm were excluded to ensure the consistency of the predictions; (ii) the sites located within 5 Å of the catalytic center were omitted to safeguard the enzyme’s functionality (Figure S1); (iii) the sites that could potentially disrupt critical hydrogen bonds or salt bridges, which are essential for structural stability, were eliminated. As a result of this meticulous screening process, 21 candidate variants were prioritized for the experimental validation (Figure 1). The comprehensive integration of computational predictions and strict filtering criteria facilitated the identification of variants, thereby improving the thermal stability without compromising the catalytic efficiency.

**Figure 2:**
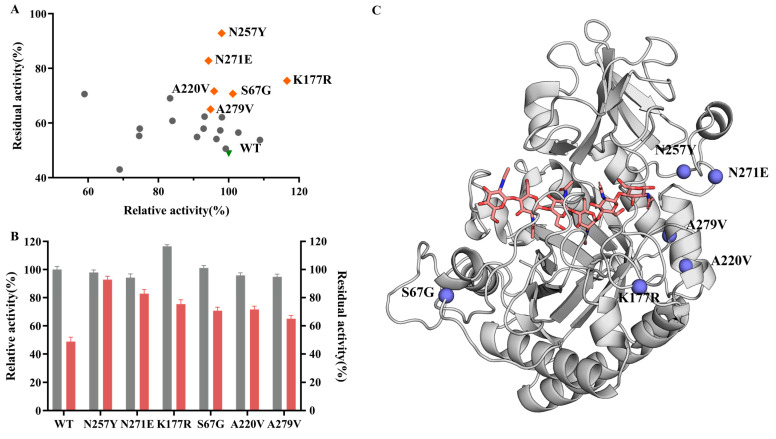
The thermal stability of BcChiA1 and its variants. (**A**) Relative activities and residual activities of single variants. Green triangle, wild type; orange diamonds, favorable variants; gray dots, unfavorable variants. The residual activities were measured after the incubation of the variants at 55 °C for 15 min. (**B**) Initial enzyme activity and residual enzyme activity of BcChiA1 and six favorable variants. Gray, initial enzyme activity; reddish, residual enzyme activity. (**C**) Schematic representation of the favorable mutation sites in BcChiA1. Violet spheres represent the favorable variants, while the substrate is displayed by light-orange sticks.

BcChiA1 and variant proteins were prepared as described (Figure S2), and the relative activity and residual activity of the variants were measured. BcChiA1 retained only 48.81% of residual enzyme activity after incubation at 55 °C for 15 min, while 20 beneficial single-point variants (except R366I) showed 51–92% residual enzyme activity. Additionally, most of the variants (except T126I and R366I) exhibited at least 70% relative enzyme activity compared to the wild type (Figure 2A; Table S3). It is noteworthy that the variants N257Y, N271E, K177R, S67G, A220V, and A279V exhibited relative enzyme activities at the WT level and retained more than 65% residual activity, with a much higher thermal stability compared to the WT (Figure 2B; Table S3). Among the six variants, N257Y, N271E, K177R, and S67G were located in flexible loop regions, while A220V and A279V were situated in the rigid helical region (Figure 2C). By simultaneously modifying sites in both the flexible and rigid regions, a synergistic effect on enzymatic properties may be achieved [41]. Therefore, these six variants were selected for combinational mutagenesis.

### 3.2. Combining Mutations to Enhance the Thermostability of BcChiA1

Previous studies reported that the combination of single beneficial mutations synergetically enhanced the thermal stability of the proteins [42,43]. Here, the combined variants were constructed with N257Y (referred as Mu1) as a starting point, which exhibited the highest residual enzyme activity without sacrificing the relative activity (Figure 2A). We then introduced N271E, K177R, S67G, A220V, and A279V into the variant Mu1 using a stepwise combination strategy.

**Figure 3:**
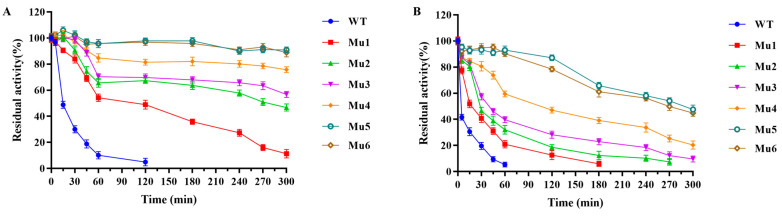
Evaluation of the thermal stability of BcChiA1 and the combined variants. (**A**) Thermostability of variants at 55 °C. (**B**) Thermostability of variants at 60 °C.

The combined variants had enzymatic activity at the WT level (Table 1), but maintained more than 50% of the residual enzyme activity after 120 min of heat treatment at 55 °C, which was significantly higher than that of the WT (which only maintained nearly 5% of the residual enzyme activity) (Figure 3A). Notably, the combined variants Mu5 and Mu6 retained nearly 90% of the residual enzyme activity after 300 min of heat treatment at 55 °C (Figure 3A). To further evaluate the thermal stability of these combined variants, an extended heat treatment was conducted at 60 °C. The half-life (*t*_1/2_, 60 °C) of BcChiA1 was 5 min. All of the variants showed prolonged half-lives: 13 min (Mu1), 27 min (Mu2), 42 min (Mu3), 116 min (Mu4), 295 min (Mu5), and 275 min (Mu6). Notably, the half-life (*t*_1/2_, 60 °C) of Mu5 was 59 times higher than that of BcChiA1 (Figure 3B; Table 1). However, the introduction of A279V into the variant Mu5 did not further enhance the thermal stability of Mu6, which may have resulted from the epistasis effect [44]. Probably, the A279 site was near to the N271 site, and these two sites may influence each other.

Previous studies on native chitinases indicated that these enzymes typically exhibited poor stability under thermal stress [45,46,47]. In recent years, various strategies have been employed to enhance the thermal stability of chitinases. For the chitinase from *Paenibacillus pasadenensis* CS0611 (PpCi1), by introducing disulfide bonds and a proline substitution, the half-life of the variant (S244C-I319C/T259P) at 50 °C was extended to 150 min [18]. Similarly, the chitinase from *Serratia marcescens* B4A, carrying the G191V mutation, exhibited a 50% decrease in enzyme activity after 90 min of incubation at 60 °C [17]. Here, the Mu5 variant demonstrated a much more favorable thermal stability, with a half-life of 295 min at 60 °C, maintaining significantly higher enzyme activity at elevated temperatures.

The *T*_m_ values of BcChiA1 and the variants were determined by using DSF. The results showed that the *T*_m_ value of the wild type was 50 °C, whereas the variants exhibited a notable increase in thermal stability. Specifically, the *T*_m_ values of Mu1, Mu2, Mu3, Mu4, Mu5, and Mu6 were 4, 6, 6, 7, 10, and 10 °C higher, respectively, indicating a significant enhancement in thermal resistance across the variants (Table 1 and Figure S3).

Among the six combined variants, Mu5 showed the most outstanding performance, which had higher relative enzyme activity and longer half-life at 60 °C than the others. These results clearly demonstrate the effectiveness of the combinatorial strategies employed in this study, which successfully generated a small but highly efficient library of variants with improved thermostability.

### 3.3. Determination of Kinetic Parameters and Enzymatic Properties of BcChiA1 and Variant Mu5

**Figure 4:**
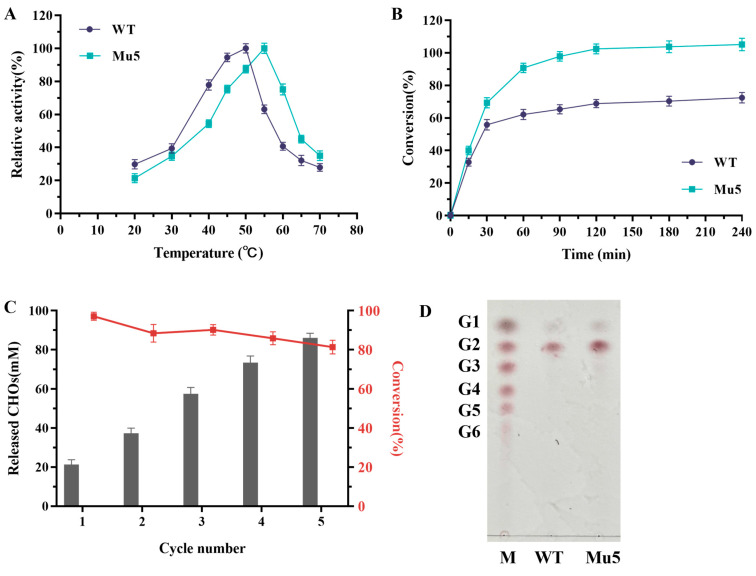
Comparison of enzymatic properties between BcChiA1 and the variant Mu5. (**A**) The optimal temperature of BcChiA1 and Mu5. (**B**) Time course of colloidal chitin hydrolysis by the WT and Mu5. (**C**) Reusability of Mu5 (the reaction was carried out at 50 °C for 90 min). (**D**) Analysis of hydrolysis products by TLC. M, G1-G6 represent GlcNAc, (GlcNAc)_2_, (GlcNAc)_3_, (GlcNAc)_4_, (GlcNAc)_5_, and (GlcNAc)₆, respectively.

To comprehensively understand the enzymatic characteristics of variant Mu5, its optimal temperature, kinetic parameters, and hydrolytic performance were evaluated. The hydrolysis reactions were conducted across a temperature range from 20 to 70 °C to assess the changes in the optimal temperature. As shown in Figure 4A, the wild-type BcChiA1 exhibited peak activity at 50 °C, whereas Mu5 displayed an elevated optimal temperature of 55 °C, representing a 5 °C increase (Figure 4A). This enhancement highlighted the potential of Mu5 for applications requiring greater thermal resilience.

The kinetic parameters of the BcChiA1 and variant Mu5 for colloidal chitin were determined under optimal conditions (Table 2 and Figure S4). The maximum reaction rate (*V*_max_) of BcChiA1 was 28.63 µmol·min^−1^·mg^−1^, the *K*_m_ value was 5.31 mg·L^−1^, and the *k*_cat_ value of BcChiA1 was 9.54 s^−1^, with a *k*_cat_/*K*_m_ ratio of 1.80 mL·mg^−1^·s^−1^. The *K*_m_ values of Mu5 were comparable to those of BcChiA1, but with a higher specific activity of 23.42 U·mg^−1^, which was a 1.30-fold increase compared to the wild type. This enhanced activity of Mu5 can be attributed to its improved thermal stability, which allows the enzyme to retain its structural integrity and catalytic efficiency at elevated temperatures.

To evaluate the potential in the industrial application of Mu5, the conversion rate of BcChiA1 and the variant Mu5 was investigated at 50 °C, with 1% colloidal chitin as the substrate. Notably, BcChiA1 exhibited a rapid decline in activity after 30 min, achieving only a 65% substrate conversion rate within 90 min. In comparison, Mu5 demonstrated superior catalytic performance, reaching a conversion rate of 97% over the same period (Figure 4B). The chitinase from *Salinivibrio* sp. BAO 1801 was used to hydrolyze colloidal chitin for 8 h, resulting in an overall yield of approximately 80% [48]. Here, Mu5 achieved a more efficient conversion of colloidal chitin in a shorter time. This improvement is likely due to the increased thermal stability of Mu5, which enables it to maintain higher catalytic activity during the reaction, thereby accelerating the hydrolysis of the substrate. To further confirm the practicality of Mu5, its stability and reusability were systematically investigated over multiple reaction cycles. After five cycles, the concentration of CHOs released by Mu5 reached 86.14 ± 3.73 mM, which was 4.08 times of that yielded in the first cycle. Furthermore, the conversion rate on the substrate was still above 80% in the fifth cycle (Figure 4C). These results indicated that thermostable Mu5 can be reused to generate CHOs.

The hydrolysis products generated by BcChiA1 and the Mu5 variant were analyzed by using TLC. The analysis showed that both enzymes primarily produced chitobiose (Figure 4D), indicating that the introduced mutations did not alter the overall degradation pattern. In summary, these results emphasize that the enhanced thermostability of Mu5 significantly amplified its potential for industrial applications. In the future, immobilization could be employed to fix Mu5 onto specific carriers for sustainable large-scale chitin degradation.

### 3.4. Assessing the Stability of Mu5 by MD Simulations

**Figure 5:**
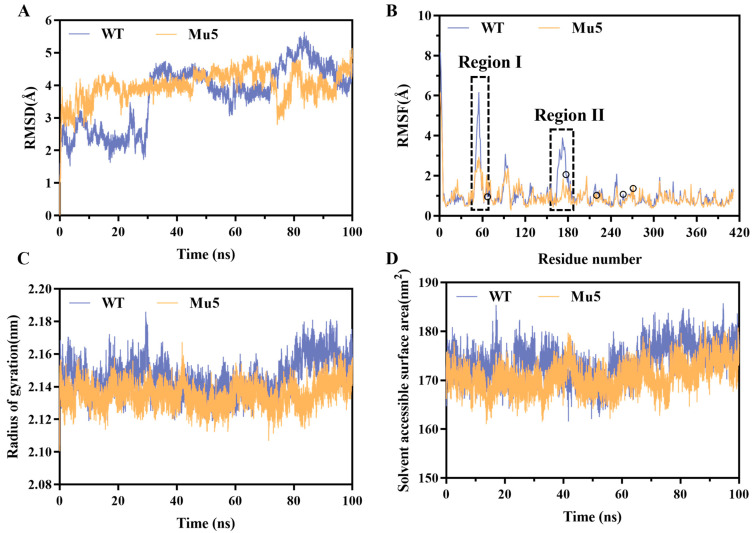
MD simulation analysis on BcChiA1 and Mu5. (**A**) RMSD plots. (**B**) RMSF plot. The five mutation sites were marked as circles. (**C**) *R*_g_ plot. (**D**) SASA plot.

Using the crystal structure of the catalytic domain of  BcChiA1 (PDB ID: 1ITX) as a template, the structure of Mu5 was modeled using AlphaFold2. The best model revealed that 95% of the residues were placed in highly reliable regions (Figure S5). To elucidate the molecular mechanism underlying the enhanced thermostability conferred by the mutations, MD simulations were conducted on BcChiA1 and Mu5 at 338 K. During the 100 ns MD simulation, the RMSD value of BcChiA1 exhibited significant fluctuations, ranging from 0.89 to 5.65 Å, whereas Mu5 maintained a relatively stable RMSD of approximately 3.89 Å (Figure 5A). The RMSD provides insights into the backbone atom movements. Mu5 maintained a more consistent ensemble of conformations with reduced transitions between different structural states, indicating an enhanced structural stability. In contrast, the WT exhibited greater conformational variability, particularly, a structural transition event around 30 ns, which may be associated with local conformational rearrangements in the flexible loop regions and a partial unfolding of specific secondary structure elements (Figure S6). The RMSF values indicate the flexibility of amino acid residues throughout the simulation, providing a detailed understanding of local structural fluctuations [49]. Notably, Mu5 demonstrated a marked reduction in the RMSF values in the following two key regions: region I (residues 51−57) and region II (residues 164−185) (Figure 5B and Figure S7A). The decreased flexibility in these regions contributed to the enhanced structural rigidity, thereby stabilizing the overall structure.

Additionally, the radius of gyration (*R*_g_) is a widely used parameter to evaluate protein structural compactness, where a lower *R*_g_ value generally correlates with increased protein stability [50]. The variant Mu5 displayed a lower *R*_g_ value (2.13 ± 0.01 nm) with minimal fluctuation, while the wild type exhibited a higher *R*_g_ value (2.15 ± 0.01 nm) and greater fluctuations (Figure 5C). Statistical analysis confirmed that this difference was significant (*p* < 0.05), suggesting that Mu5 maintained a more compact and stable structure during the simulation. To further validate the enhanced compactness of Mu5, solvent-accessible surface area (SASA) analysis was performed. The Mu5 variant exhibited a lower average SASA (170.61 ± 2.89 nm^2^) compared to the wild type (173.76 ± 3.50 nm^2^) over the entire MD simulation (*p* < 0.05) (Figure 5D). This structural compactness is typically associated with a higher structural stability, as it reduces the entropy of protein unfolding and helps to maintain the folded state under thermal stress [51].

In summary, the enhanced thermal stability of the Mu5 variant can be attributed to a combination of multiple factors, including reduced structural flexibility and an increased compactness of its tertiary structure. These factors collectively enabled the Mu5 variant to preserve structural integrity under elevated temperatures, thereby offering greater resistance to thermal denaturation compared to the wild-type protein.

### 3.5. Structural Investigations on Variant Mu5

To further elucidate the potential molecular mechanisms underlying the enhanced thermal stability of the Mu5 variant, an in-depth analysis of the interaction changes was conducted.

In this study, the mutation of Ser67 to Gly67 led to the formation of a new hydrogen bond between Gly67 and Asn64 (Figure S8A). The K177R variant introduced a longer arginine side chain with an additional guanidinium group, allowing Arg177 to form a hydrogen bonding network with the surrounding residues, including Ser169, Gly171, Leu172, and Asn175 (Figure S8B). Notably, the introduction of Gly67 and Arg177 also induced the formation of hydrogen bonds between Asp173 and Asp51, further stabilizing the local structure (Figure S7B,C). The RMSF values from the MD simulation revealed that the variations significantly stabilized the tertiary structure of region I (residues 51−57) and region II (residues 164−185) (Figure 5B). Similarly, in aldehyde dehydrogenase (ADA6) from *Buttiauxella* sp. S04-F03, the introduction of the P443C and P253V mutations increased the frequency of hydrogen bond formation in the E433-T453 and P243-A263 regions. These introduced interactions effectively stabilized the local structural regions, leading to a significant enhancement in the overall thermal stability of ADA6 [52]. This further supports the critical role of local structural stability in enhancing protein thermal stability [53]. Although the substitution of Asn271 by Glu271 did not introduce new electrostatic interactions or hydrogen bonds, the negative charge of the glutamic acid residue significantly increased the polarity of the protein surface (Figures S8C and S9A), thereby improving the solubility of the protein in an aqueous environment [54]. This increase in solubility helped to reduce protein aggregation at high temperatures and further improved the thermal stability of proteins [55].

The pathways of protein folding are significantly influenced by hydrophobic interactions that arise between hydrophobic residues [23,53]. In this study, Ala220 was located on the surface of proteins and participated in the formation of hydrophobic clusters with Leu232, Thr230, and Ala224 (Figure S8D). The introduction of Val220 enhanced local hydrophobic interactions, further stabilizing the structure. In addition, Val220 formed additional hydrogen bonds with Ala217 and Lys182, reinforcing the overall stability of the enzyme (Figure S8D). The mutation of Asn257 to Tyr257 facilitated the formation of a hydrophobic cluster with adjacent residues A276 and Y382 (Figure S8E). Moreover, the introduction of a larger aromatic side chain at Asn257 effectively filled the cavity core, which contributed to the overall stability of Mu5 (Figure S9B). In summary, these mutations led to structural changes in Mu5 and significantly enhanced the overall stability of Mu5 in high-temperature environments.

## 4. Conclusions

In this study, we successfully enhanced the thermal stability of chitinase from *Bacillus circulans* WL-12 through a comprehensive computational design approach. A total of 21 candidate sites that may contribute to the enzyme’s stability were identified; then, the single variants with thermostability improvement were applied for combination. The penta-variant Mu5 exhibited a significant extension of the half-life (from 5 min to 295 min) at 60 °C. The thermostable Mu5 demonstrated promising performance in the depolymerization of colloidal chitin. The MD simulations revealed that the enhanced thermostability was primarily driven by increased structural rigidity and compactness, which reduced the enzyme’s susceptibility to thermal denaturation. This work generated a robust chitinase variant for industrial applicability. The strategies employed here could be extended to other enzymes, providing a pathway for generating more robust and efficient biocatalysts for a variety of industrial processes.

## Figures and Tables

**Table 1:** Relative enzymatic activity, *T*_m_, and *t*_1/2_ values of BcChiA1 and the variants.

	Mutation	Relative Activity (%)	*T*_m_ (°C)	*t*_1/2_ (min) *
WT	wild type	100 ± 2.28	50 ± 0.3	5
Mu1	N257Y	96 ± 1.88	54 ± 0.4	13
Mu2	N257Y/N271E	98 ± 2.73	56 ± 0.2	27
Mu3	N257Y/N271E/K177R	105 ± 1.98	56 ± 0.5	42
Mu4	N257Y/N271E/K177R/S67G	101 ± 2.83	57 ± 0.3	116
Mu5	N257Y/N271E/K177R/S67G/A220V	109 ± 2.08	60 ± 0.4	295
Mu6	N257Y/N271E/K177R/S67G/A220V/A279V	106 ± 3.22	60 ± 0.5	275

* The half-lives of BcChiA1 and the variants measured at 60 °C.

**Table 2:** Kinetic parameters of BcChiA1 and Mu5.

	*V*_max_ (µmol·min^−1^·mg^−1^)	*K*_m_ (mg/L)	*k*_cat_ (s^−1^)	*k*_cat_*/K*_m_ (mL·mg^−1^·s^−1^)	Specific Activity (U/mg)
BcChiA1	28.63 ± 1.89	5.31 ± 0.53	9.54 ± 0.64	1.80 ± 0.22	17.93 ± 2.32
Mu5	30.74 ± 2.03	5.02 ± 0.69	10.25 ± 0.68	2.04 ± 0.31	23.42 ± 2.03

## Data Availability

The original contributions presented in the study are included in the article and Supplementary Materials; for further inquiries, please contact the corresponding author.

## Supplementary Materials

The following supporting information can be downloaded at: https://www.mdpi.com/article/10.3390/biom15030330/s1, Figure S1: The binding pocket of chitinase A1 (PDB ID: 1ITX) with NAG_6_; Figure S2: SDS-PAGE analysis of the wild type and variants after purification from nickel columns; Figure S3: Measurement of the melting temperature (*T*_m_) of the WT and combined variants; Figure S4: Michaelis–Menten curve of the WT and Mu5. Figure S5: The best structural model of the catalytic domain in Mu5 generated by AlphaFold2; Figure S6: Comparison of structures of the WT and Mu5 before and after the heat treatment; Figure S7: Interaction analysis of region I and II in the structures of the WT and Mu5; Figure S8: Interaction analysis on mutation sites in Mu5; Figure S9: The electrostatic potential energy and surface structure analysis of BcChiA1 and Mu5; Table S1: Primer sequences for site-directed mutagenesis; Table S2: Computational prediction of potential mutation sites; Table S3: Enzyme activity and thermal stability of the WT and variants.
