# Supplementary material for: Thermostability Improvement of the Chitinase from Bacillus circulans for Efficient Chitin Oligosaccharide Production via Computational Design
Source: Biomolecules. 2025 Feb 24;15(3):330. doi: 10.3390/biom15030330 (PMC11940100; doi:10.3390/biom15030330)
Supplement: Supplementary file 1 [file biomolecules-15-00330-s001.zip › biomolecules-3458800-supplementary.pdf]

Supplementary Information

Thermostability Improvement of the Chitinase from *Bacillus circulans* for Efficient Chitin Oligosaccharides Production via Computational Design

Jingwei Liu[1,2,3], Jie Xie[1,3], Si Wang[1,3], Hong Feng[2] and Ganggang Wang[1]*

1 Key Laboratory of Environmental Microbiology of Sichuan Province; Key Laboratory of Environmental and Applied Microbiology of Chinese Academy of Sciences, Chengdu Institute of Biology, Chinese Academy of Sciences, Chengdu 610041, China

2 College of Life Sciences, Sichuan University, Chengdu 610064, China

3 University of Chinese Academy of Sciences, Beijing 100049, China

*To whom correspondence should be addressed:

Prof. Ganggang Wang

Key Laboratory of Environmental and Applied Microbiology, Chengdu Institute of Biology, Chinese Academy of Sciences, Chengdu, 610041, China

Tel.: +86-28-82890828; Fax: +86-28-82890828; E-mail: wanggg@cib.ac.cn

Table S1. Primer Sequences for Site-Directed Mutagenesis

Variants	Primers	Sequences (5'→3')
S67G	S67G-F	CTGTCAGAATGAAAAAGGCCAAACGATCAATG
	S67G-R	CATTGATCGTTTGGCCTTTTTCATTCTGACAG
T126I	T126I-F	GTTGGAGGATGGATCTGGTCCAACCGTTTCTC
	T126I-R	GAAACGGTTGGACCAGATCCATCCTCCAACG
D173P	D173P-F	GTATCAGGCGGACTCCCTGGTAACAGCAAACG
	D173P-R	GTTTGCTGTTACCAGGGAGTCCGCCTGATACCG
K177R	K177R-F	CTCGATGGTAACAGCAGACGTCCTGAAGATAAG
	K177R-R	CTTATCTTCAGGACGTCTGCTGTTACCATCGAG
Y185F	Y185F-F	CCTGAAGATAAGCAAAACTTCACATTGCTCTTG
	Y185F-R	GCTCAAGAGCAATGTGAAGTTTTGCTTATCTTC
S190Q	S190Q-F	CACATTGCTCTTGCAGAAAATCCGTGAAAAG
	S190Q-R	CTTTTCACGGATTTTCTGCAAGAGCAATGTG
V202Q	V202Q-F	GCAGCGGGAGCTCAGGACGGCAAGAAGTATC
	V202Q-R	GATACTTCTTGCCGTCCTGAGCTCCCGCTGC
T218D	T218D-F	CGGTGCGTCTGCGGACTATGCTGCCAATACGGAG
	T218D-R	CTCCGTATTGGCAGCATAGTCCGCAGACGCACCG
A220V	A220V-F	CTGCGACCTATGTCGCCAATACGGAGCTTGC
	A220V-R	GCAAGCTCCGTATTGGCGACATAGGTCGCAG
N257Y	N257Y-F	CATAATGCGCCGTTGTACTATGATCCTGCG
	N257Y-R	CGCAGGATCATAGTACAACGGCGCATTATG
D269N	D269N-F	CTGGCGTGCCAAACGCCAATACATTTAATG
	D269N-R	CATTAAATGTATTGGCGTTTGGCACGCCAG
N271E	N271E-F	GCTGGCGTGCCAGATGCCGAGACATTTAATGTGGC
	N271E-R	CACATTAAATGTCTCGGCATCTGGCACGCCAGCGGC
A279V	A279V-F	CATTTAATGTGGCTGCCGGAGTACAAGGGCATTTG
	A279V-R	CAAATGCCCTTGTACTCCGGCAGCCACATTAAATG
V296I	V296I-F	CTAAACTTGTGCTTGGTATTCCATTCTATGGCC
	V296I-R	GGCCATAGAATGGAATACCAAGCACAAGTTTAG
A307P	A307P-F	GCTGGGATGGATGCCCACAGGCAGGCAACGGCC
	A307P-R	GCCGTTGCCTGCCTGTGGGCATCCATCCCAGCC
F333Y	F333Y-F	CAGGATCCTTTGACTACTATGATCTGGAAGC
	F333Y-R	GCTTCCAGATCATAGTAGTCAAAGGATCCTG

(Continue) Table S1. Primer Sequences for Site Directed Mutagenesis

Variants	Primers	Sequences (5’→3’)
F333Y	F333Y-F	CAGGATCCTTTGACTACTATGATCTGGAAGC
	F333Y-R	GCTTCCAGATCATAGTAGTCAAAGGATCCTG
T353I	T353I-F	GTTACTGGAATGACATCGCCAAAGTGCCATATC
	T353I-R	GATATGGCACTTTGGCGATGTCATTCCAGTAAC
A362P	A362P-F	GCCATATCTCTATAATCCGTCCAATAAGCGC
	A362P-R	GCGCTTATTGGACGGATTATAGAGATATGGC
R366I	R366I-F	GCGTCCAATAAGATCTTTATCAGTTATG
	R366I-R	CTGATAAAGATCTTATTGGACGCATTATAG
A373E	A373E-F	CTTTATCAGTTATGACGATGAGGAGTCCGTTGG
	A373E-R	GTTTTATATCCAACGGACTCCTCATCGTCATAACTG
A391V	A391V-F	CAAAGGACTCGGCGGAGTGATGTTCTGGGAGC
	A391V-R	GCTCCCAGAACATCACTCCGCCGAGTCCTTTG

Note: Mutated codons are highlighted in red and underlined

Table S2. Computational Prediction of Potential Mutation Sites

PROSS	FireProt 2.0	ABAUCS	detrimental effects	selected for experiments
G9A			predicted by only one software	
	T29S	T29S	hydrogen bonds were broken	
I46R			predicted by only one software	
**S67G**	**S67G**	**S67G**		**S67G**
	N104F	N104F	hydrogen bonds were broken	
		T118I	predicted by only one software	
**T126I**	**T126I**			**T126I**
R130Y		R130Y	hydrogen bonds were broken	
	D150W		predicted by only one software	
**D173P**	**D173P**	**D173P**		**D173P**
**K177R**		**K177R**		**K177R**
**Y185F**		**Y185F**		**Y185F**
**S190Q**	**S190Q**	S190K		**S190Q**
**V202Q**		**V202Q**		**V202Q**
S213A		S213A	hydrogen bonds were broken	
A217P		A217P	proline was unfavorable to α-helix stability	
**T218D**		**T218D**		**T218D**
A220I	**A220V**	**A220V**		**A220V**
A245S			predicted by only one software	
**N257Y**	**N257Y**	**N257Y**		**N257Y**
		S263L	predicted by only one software	
**D269N**		**D269N**		**D269N**
**N271E**		**N271E**		**N271E**
**A279V**		**A279V**		**A279V**
	G281M	G281M	hydrogen bonds were broken	
D284N			predicted by only one software	
**V296I**		**V296I**		**V296I**
**A307P**		**A307P**		**A307P**
		G320P	predicted by only one software	
		D332L	predicted by only one software	
**F333Y**	**F333Y**	**F333Y**		**F333Y**
**T353I**		**T353I**		**T353I**
A362P	**A362R**	**A362R**		**A362R**
**R366I**		**R366Y**	hydrogen bonds were broken	**R366I**
**A373E**		**A373E**		**A373E**
**A391V**	A391T	**A391V**		**A391V**
E395D		E395Y	within 5 Å distant from the ligand	
Q405L		Q405L	hydrogen bonds were broken	

Note: The sites selected for experiments were highlighted in bold.

Table S3. Enzyme Activity and Thermal Stability of WT and Variants

Enzyme	Relative enzyme activity (%)	Residual enzyme activity (%)
WT	100±2.33	48.81±2.69
S67G	101.15±3.06	70.71±1.95
T126I	58.98±3.33	70.65±2.27
D173P	83.98±3.25	60.82±2.34
K177R	116.55±3.58	75.46±2.73
Y185F	102.73±2.83	56.51±1.88
S190Q	74.69±3.47	57.97±2.49
V202Q	97.59±2.69	57.30±3.64
T218D	96.50±3.72	54.17±3.05
A220V	95.82±2.59	71.67±2.48
N257Y	97.97±3.05	92.86±2.55
N271E	94.22±2.61	82.80±3.36
D269N	93.09±3.73	62.35±2.69
A279V	94.82±3.43	65.10±3.57
V296I	98.05±2.88	62.14±2.49
A307P	83.32±2.83	69.01±3.38
F333Y	90.96±2.57	54.93±3.61
T353I	99.13±2.17	50.62±3.54
A362P	74.53±3.47	55.34±2.84
R366I	68.97±3.33	43.04±2.59
A373E	108.86±2.25	53.80±3.07
A391V	92.82±3.42	57.96±2.18

Note: The variants selected for combinational mutagenesis were highlighted in red.

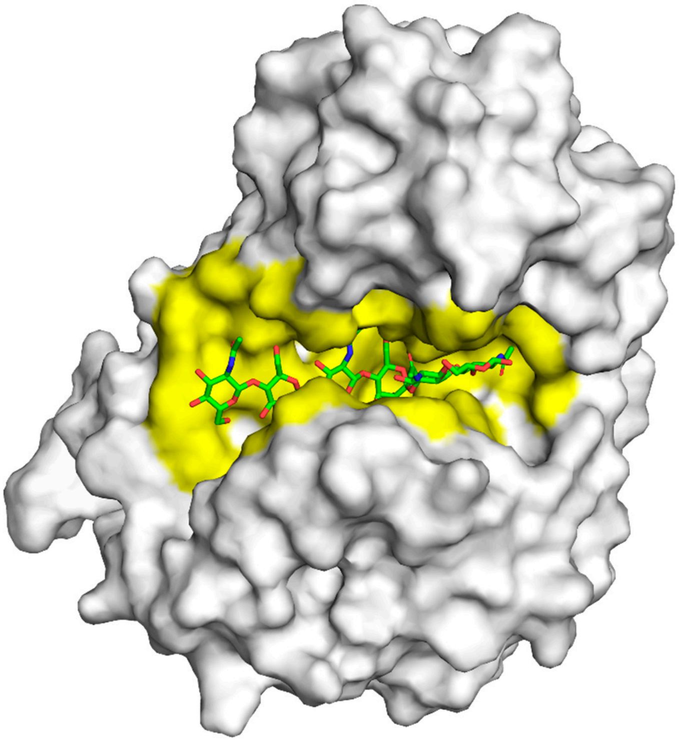

Figure S1. The binding pocket of chitinase A1 (PDB: 1ITX) with NAG_6. The amino acid residues within 5 Å of NAG_6 are displayed in yellow.

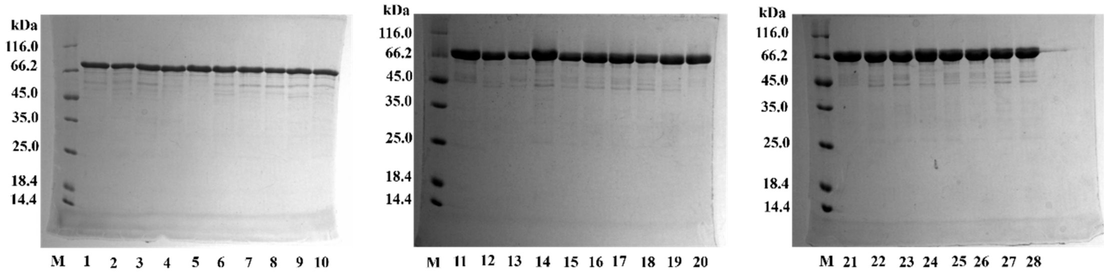

Figure S2. SDS-PAGE analysis of wild type and variants after purification from nickel columns. Lanes 1-28 in order of WT, S67G, T126I, D173P, K177R, Y185F, S190Q, V202Q, T218D, A220V, N257Y, N271E, D269N, A279V, V296I, A307P, F333Y, T353I, A362P, R366I, A373E, A391V, Mu1, Mu2, Mu3, Mu4, Mu5, Mu6. M: Marker.

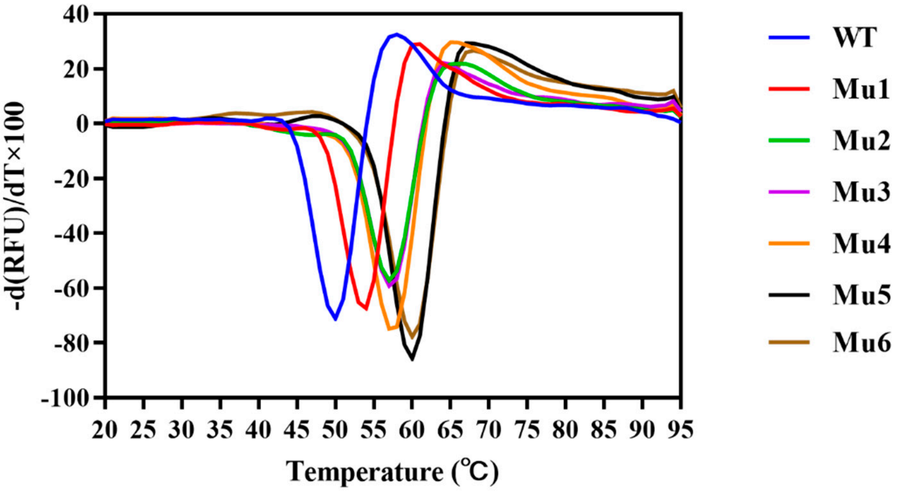

Figure S3. Measurement of the melting temperature (T_m) of WT and combined variants.

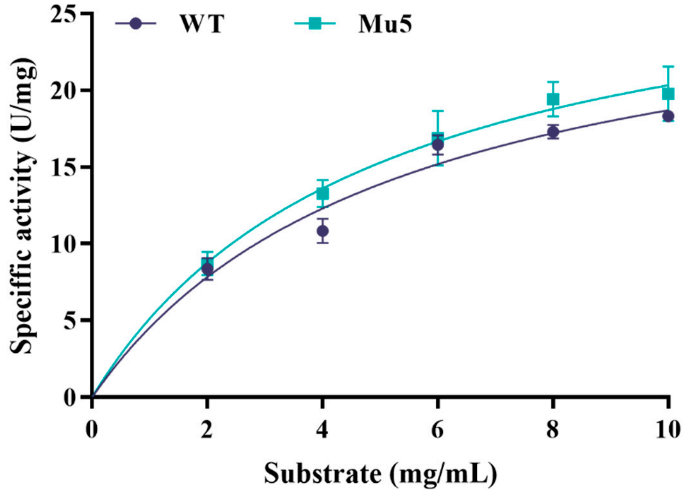

Figure S4. Michaelis-Menten curve of WT and Mu5.

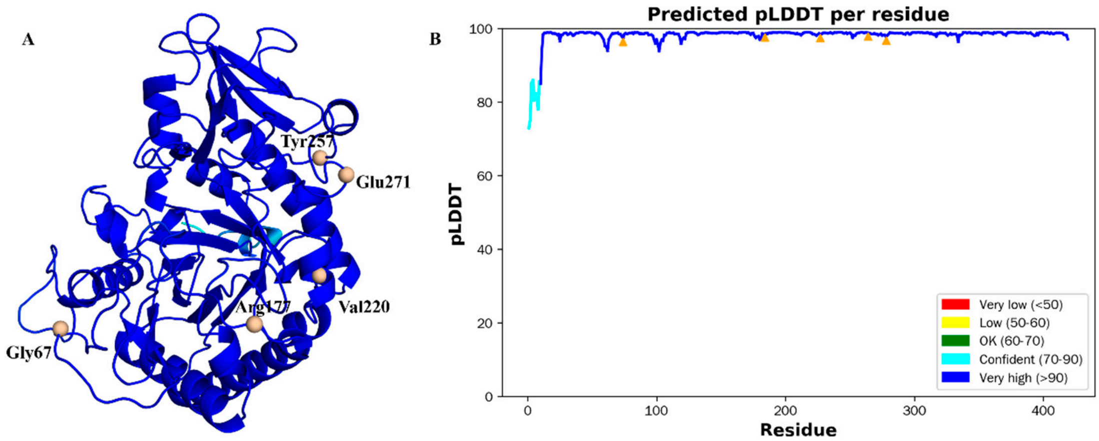

Figure S5. The best structure model of the catalytic domain in Mu5 generated by AlphaFold2 (A) and the quality assessment of the predicted model (B). In panel A, the protein structure is colored based on the predicted pLDDT values to indicate the confidence level of different regions. The light-yellow sphere represents the favorable variants. In panel B, the mutation sites were marked as orange triangle.

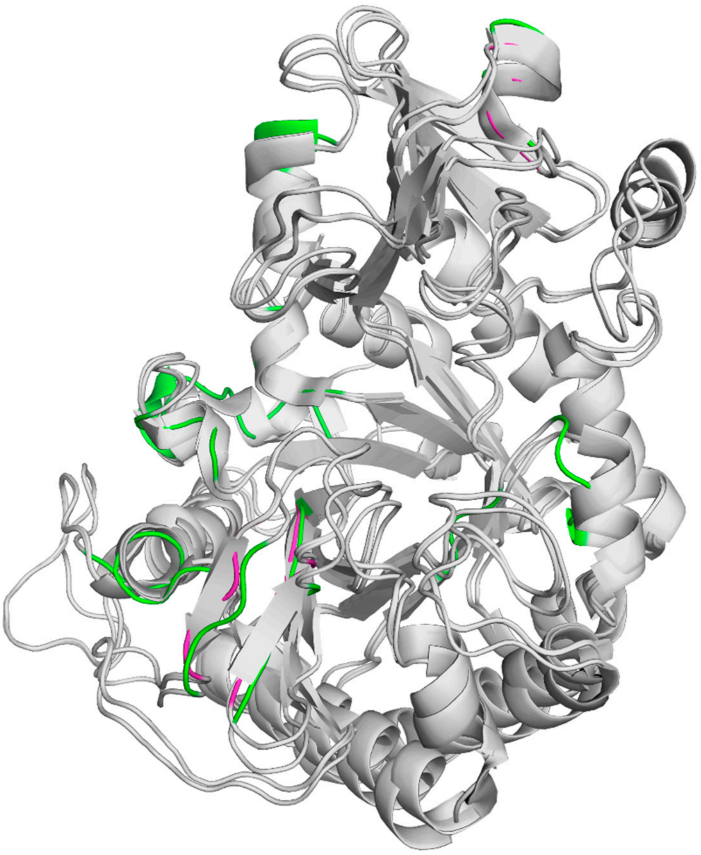

Figure S6. Comparison of structures of WT and Mu5 before and after heat treatment. Green, changes in WT structure; pink, changes in Mu5 structure.

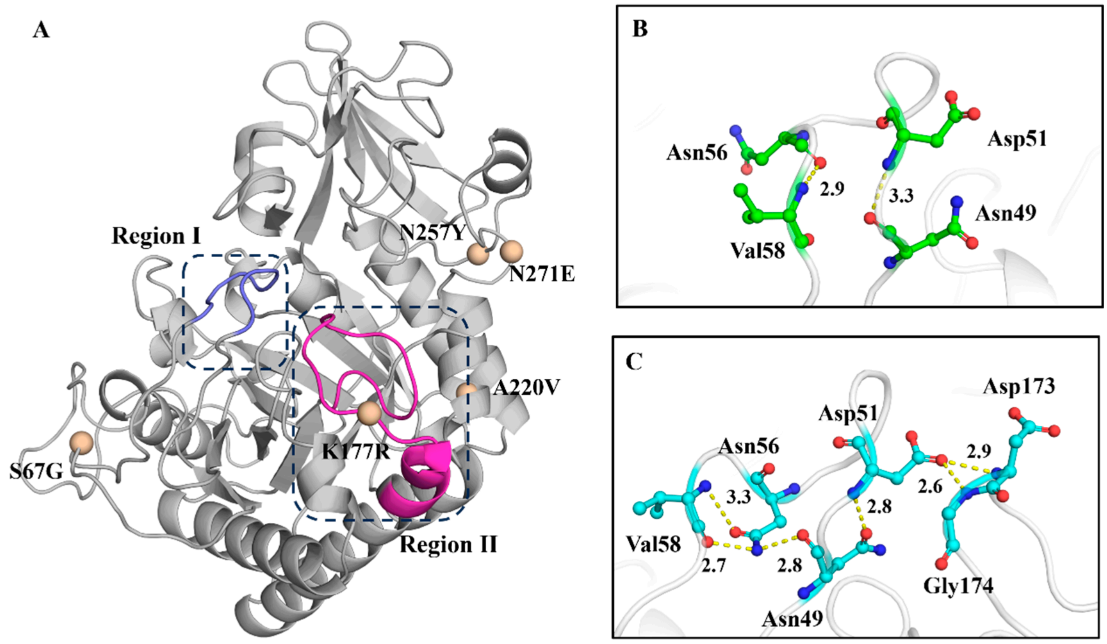

Figure S7. Interaction analysis of region I and II in the structures of WT and Mu5. (A) Corresponding positions of region I and II in the three-dimensional structure of BcChiA1. Region I and II are displayed as cartoon in purple and pink, the light-yellow spheres represent the favorable variants, respectively. (B) Interaction analysis of region I and II in WT. (C) Interaction analysis of region I and II in Mu5. The yellow dashed lines represent hydrogen bonds.

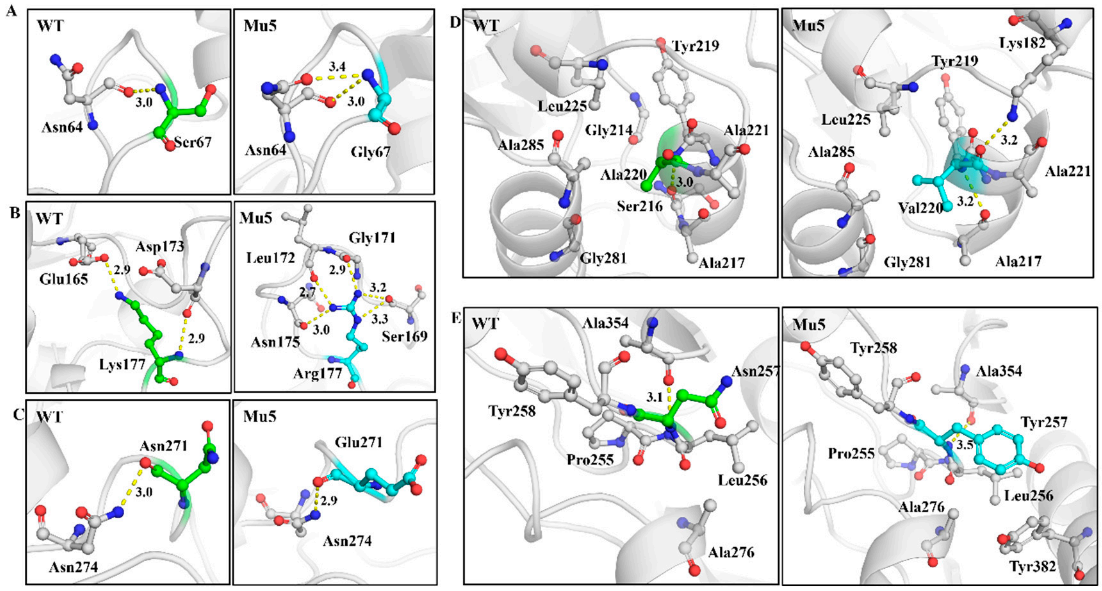

Figure S8. Interaction analysis on mutation sites in Mu5. The candidate sites in wild-type and mutated residues in Mu5 are shown as sticks in green and cyan, respectively. The other residues are shown as white sticks. The yellow dashed lines represent hydrogen bonds.

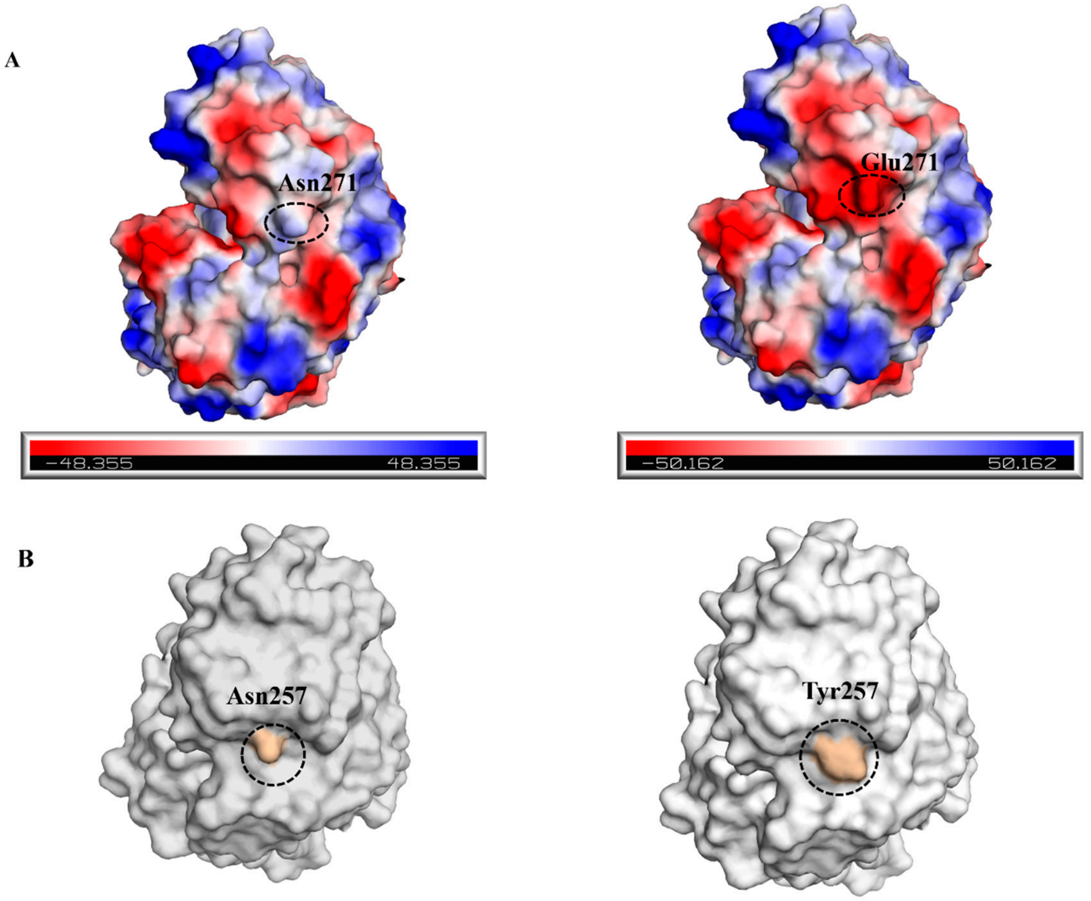

Figure S9. The electrostatic potential energy and surface structure analysis of BcChiA1 and Mu5. (A) The electrostatic potential energy of BcChiA1 (left) and Mu5 (right), the electrostatic potential energy with variant N271E of the enzyme were marked with black frames. (B) The surface structure of BcChiA1 (left) and Mu5(right), the surface structure with variant N257Y of the enzyme were marked with black frames.
